# Understanding and Exacerbating the Biological Response of Uveal Melanoma to Proton Beam Therapy

**DOI:** 10.3390/cancers17193104

**Published:** 2025-09-24

**Authors:** Laura Hawkins, Helen Kalirai, Karen Aughton, Rumana N. Hussain, Sarah E. Coupland, Jason L. Parsons

**Affiliations:** 1 Institute of Cancer and Genomic Sciences, University of Birmingham, Edgbaston, Birmingham B15 2TT, UK; 2 Department of Eye and Vision Science, Institute of Life Course and Medical Science, University of Liverpool, Liverpool L7 8TX, UK; 3 Liverpool Ocular Oncology Centre, Liverpool University Hospitals Trust, Liverpool L7 8XP, UK; 4 School of Physics and Astronomy, University of Birmingham, Edgbaston, Birmingham B15 2TT, UK

**Keywords:** autophagy, DNA damage, DNA repair, hypoxia, ionising radiation, proton beam therapy, uveal melanoma

## Abstract

Uveal melanoma (UM) is the most common primary intraocular malignancy in adults, associated with a high tendency for metastasis to the liver. Proton beam therapy (PBT) is the preferred external radiotherapy treatment for primary UM of certain sizes and locations in the eye, due to its efficacy and good local tumour control, as well as its precision to spare surrounding ocular structures. PBT is an effective alternative to surgical enucleation and other non-precision-targeted radiotherapies. Despite this, the radiobiology of UM in response to PBT is still not fully understood. This enhanced knowledge would help to further optimise UM treatment and improve patient outcomes through reducing radiation dosage to ocular structures, treating larger tumours that would otherwise require enucleation, or even offering a treatment strategy for the otherwise fatal liver metastases. In this review, we explore current knowledge of the treatment of UM with PBT, evaluating the biological responses to the therapy. Molecular factors, such as tumour size, oxygen tension levels, DNA damage proficiency, and autophagy, are known to influence the cellular response to radiotherapy, and these will be discussed. Furthermore, we examine innovative strategies to enhance radiotherapy outcomes, such as combination therapies with DNA damage repair and autophagy modulators, as well as advancements in PBT planning and delivery. By integrating current research and emerging technologies, we aim to provide opportunities to improve the therapeutic effectiveness of PBT in UM management.

## Simple Summary

Proton beam therapy is a well-established precision radiotherapy treatment for cancers of the eye (uveal melanoma) that minimises damage to the surrounding normal tissues and preserves vision. The treatment effectiveness stems from the induction of DNA damage in tumour cells, which can overwhelm the repair mechanisms, driving cellular death. Despite the success of proton therapy, a subset of patients exhibit suboptimal responses, with some experiencing local recurrence and/or development of metastasis. This review presents current evidence on the biological factors, such as DNA repair mechanisms and reduced oxygen (hypoxia), that drive the responses of uveal melanoma to proton therapy. We also discuss emerging strategies aimed at treatment effectiveness, including targets for combination therapies. Understanding these aspects is critical for optimising treatment outcomes for eye cancer patients.

## 1. Introduction

Uveal melanoma (UM) is a rare and aggressive malignancy that originates from transformed melanocytes in the eye. It is the most common primary intraocular cancer in adults, with approximately 2–8 cases per million population per year [1,2]. Approximately 50% of UM patients will eventually develop metastases, most commonly to the liver, with a median survival of around 4–7 months once metastatic disease is detected [3,4]. The survival of UM patients with metastases has not greatly improved over the last three decades, with recent clinical trials involving chemotherapies and immunotherapies showing limited clinical efficacy and severe adverse effects [2,5,6]. Standard-of-care treatment for primary UM is typically radiotherapy administered as ocular brachytherapy, involving the surgical placement of a radioisotope plaque onto the tumour site, if the size and location of the tumour permit. Alternatively, the primary tumour may be treated with proton beam therapy (PBT), an external form of radiation [7,8]. Stereotactic radiosurgery, Gamma Knife and CyberKnife have also been shown to be effective; however, they are reportedly associated with higher rates of adverse effects to other ocular structures [9]. Local surgical resection of the tumour can be performed with the eye remaining intact; however, this is performed only on a small percentage of UM patients and only in some ocular oncology centres [10]. Otherwise, surgical removal (enucleation) of the eye is still performed regularly; this causes permanent vision loss and often psychological, social, and emotional distress [11,12]. There is no current proven survival advantage between any of these treatment options [7,13].

In the UK, approximately 70% of UM patients receive radiotherapy as their initial treatment, with 40–50% receiving Ruthenium-106 plaque brachytherapy and between 20 and 40% of patients treated with PBT [14]. The remaining 30% of patients undergo enucleation. Treatment trends for UM patients in the UK varies depending on which of the four ocular oncology centres are managing the case. These centres are located in Sheffield, Glasgow, London, and Liverpool, and treatment decisions depend on surgeon preference, experience, and accessibility [15]. The Clatterbridge Cancer Centre in Liverpool is home to the UK’s only Eye PBT centre, which has been delivering treatment for ocular melanomas since 1989. Patients registered at the Liverpool Ocular Oncology Centre have higher use of PBT as their initial treatment compared to patients in Ireland, a reflection of the increased access to this modality [15]. PBT is well-established as a core radiotherapy treatment for UM, as opposed to conventional X-ray/photon radiotherapy used to treat most other cancer types. Amongst PBT centres across the globe, an estimated 43,000 UM patients have been treated with targeted PBT [14,16]. In the US, more than 1600 patients have been treated at the Crocker Nuclear Laboratory at the University of California, San Francisco, another specialised facility for ocular radiotherapy [16]. There is a wealth of knowledge and experience in utilising PBT for UM.

Despite PBT forming a large part of the primary treatment of UM, surprisingly, the radiobiology of the tumour response to this modality is still not well understood. PBT is a precision-targeted treatment where much of the radiation dose can be delivered directly to the tumour. However, there is uncertainty regarding the biological mechanism of action and clinical response, which are influenced by the linear energy transfer (LET) of the proton beam. With PBT, there are known increases in LET at and around the Bragg peak, where the radiation dose is deposited, which influences the biological (DNA) damage created. This also affects the cellular mechanisms that respond to the damage, although these details are not well understood.

In this review, we present current knowledge surrounding the use of PBT in the treatment of UM and provide an overview of the key biological processes reported to influence treatment efficacy. By increasing our understanding of the response of UM to PBT, strategies to enhance the effectiveness of the radiotherapy treatment can be developed, with the goals being to preserve the eye and vision and to prevent tumour recurrence and metastasis.

## 2. Proton Beam Therapy

**Figure 1:**
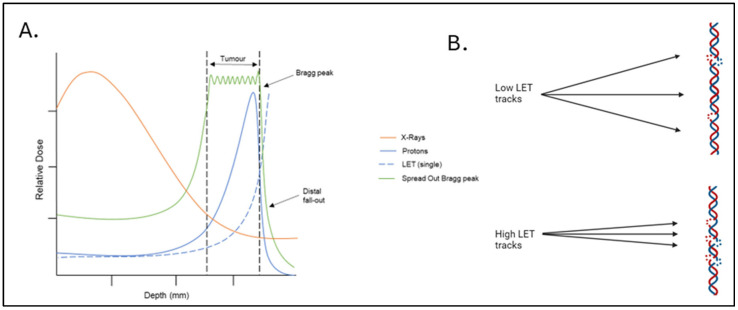
Depth–dose distribution of PBT relative to the Bragg peak and the relationship to ionisation density/LET. (**A**) PBT shows a relatively low entrance and exit dose (blue solid line), with the maximal energy being deposited in a well-defined region called the Bragg peak, that can be targeted to the tumour. This is in comparison to X-rays/photons where the dose is highest close to the source (orange line). LET (dashed blue line) increases at and around the Bragg peak. Several proton beams can be modulated and combined to create a spread-out Bragg peak (green line) to target larger tumours. (**B**) Low-LET radiation tracks cause regions of isolated DNA damage, such as NDA breaks within the tumour cell, whereas more densely ionising high-LET tracks cause regions of CDD. Created in BioRender. Hawkins, L. (2025) https://app.biorender.com/illustrations/6724f4364b0df48f0107bd27?slideId=4ed920ab-b703-4e06-a8cf-d1f8ea75740d (accessed on 13 September 2025).

PBT is a highly precise form of external beam radiotherapy that uses protons to precisely deliver ionising radiation within the tumour. PBT for UM patients is typically delivered in four fractions over four consecutive days, up to a final dose of approximately 60 Gy [17,18]. The major advantage of PBT is the accurate targeting of the tumour via dose deposition through the Bragg peak (Figure 1A). PBT delivers a low entrance and exit dose, which minimises the irradiation risk to surrounding normal tissues and structures proximal to the tumour being treated. Furthermore, the energy deposition can be modulated to a defined narrow depth, specifically targeting only the tumour and thereby reducing radiotherapy-induced side effects [19,20]. However, energy deposition via the Bragg peak also leads to an increase in LET as the protons reduce in speed and lose energy as they pass through the tissue. The increase in LET leads to a higher frequency of ionisation events along the radiation track, which, in the context of cellular DNA, can lead to elevated levels and complexity of DNA damage (Figure 1B). Complex DNA damage (CDD) is more challenging for cells to repair than isolated DNA damage, contributing to increased cell death and a greater relative biological effectiveness (RBE) [21]. Combining beams with different initial energies can create a spread-out Bragg peak (SOBP), thus allowing the targeting of tumours with large size and volume. PBT can be utilised for UM in any location in the eye and with any tumour thickness. This is a significant advantage compared to brachytherapy, which cannot be placed too close to the optic nerve [22,23,24].

## 3. Evaluating the Clinical Response to Proton Beam Therapy

There are no current prospective randomised trials that have evaluated the efficacy of PBT for UM, but there are multiple reviews and meta-analyses that demonstrate good survival rates and the safety of this treatment [25,26,27]. The most relevant reports of patient outcomes following PBT, including studies containing at least 50 patients, are summarised in Table 1. This table has been modified from Yilmaz et al., 2024 [28].

This evidence demonstrates that there is a consistent similarity in dosing protocols, with most tumours receiving a fractionated dose of 50–60 Gy RBE. PBT has been evidenced to be effective at controlling the primary tumour with reported local control rates of 91–96.5% over 5 years and 92.1–94% over 10 years [18,29,33,34]. This is coupled with good rates of eye preservation ranging from 91 to 93.2% [29,33]. Collectively, these results show that PBT is excellent at controlling the primary tumour, with high levels of local tumour control and eye retention rates. Despite this, some degree of toxicity has been reported in patients treated with PBT, although the rates are quite variable and likely attributed to tumour location and volume. It is also worth noting that some of these studies are over twenty years old. Among reported toxicities, radiation retinopathy, a common and devastating visual side effect causing disruption of vascular structures in the eye [32,33], is most frequently observed at rates of 27–74%. Secondary neovascular glaucoma has also been recognised as a side effect of PBT, with rates of 7–28%, because of the so-called ‘toxic tumour syndrome’, i.e., the release of enzymes and factors from the dying melanoma cells that induce tissue lysis as well as neovascularisation [36]. Toxic tumour syndrome often necessitates either prompt removal of the tumour via local resection or, more often, secondary enucleation [24,37]. It is, however, difficult to determine whether other complications, such as haemorrhage and retinal detachment rates, are caused by the PBT or the tumour itself.

Despite PBT being a relatively effective treatment for primary UM, as seen in Table 1, approximately 10% of patients fail in local control [31,33,34]. The rate of metastasis following treatment is up to 50%, and PBT-treated UM patients have a 74–91% survival rate for 10 years; however, these outcomes are comparable across all primary treatment methods, including enucleation and plaque brachytherapy [7,13]. This suggests that metastasis and long-term survival rates are influenced more by underlying factors such as chromosomal aberrations and genetic mutations, rather than the choice of treatment modality [38,39,40,41,42]. Importantly, PBT offers distinct advantages: it can reduce the need for enucleation, limit certain side effects such as pain, and is equally effective in treating primary tumours in terms of patient outcomes when compared to other established therapies. Research is needed to assess the variability in response to PBT, identify the underlying factors, and determine whether combinatorial treatments may improve the long-term survival of UM. Understanding the radiobiological response to PBT is therefore critical to optimising the treatment to enable good tumour control for eye and vision preservation as much as possible.

## 4. Key Molecular Factors Known to Influence Cellular Response to PBT

The radiobiological responses to PBT depend on several cellular and molecular factors, which are discussed below.

### 4.1. DNA Damage Repair Proficiency

**Figure 2:**
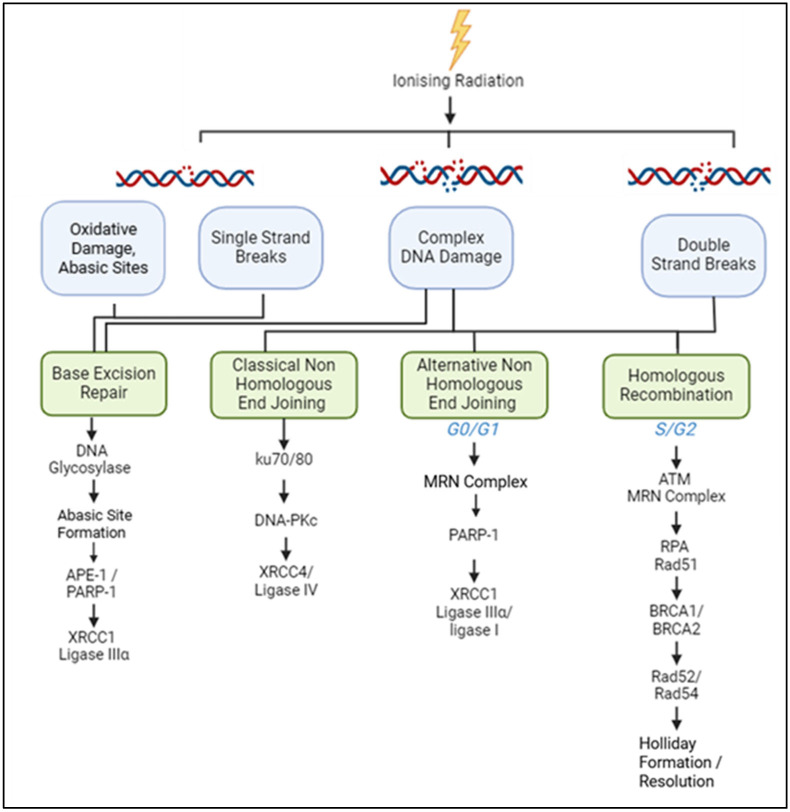
The major cellular DDR pathways responsive to PBT-induced DNA damage. Ionising radiation induces a variety of DNA lesions that are repaired by DDR pathways. SSBs and base damage are repaired by base excision repair (BER), where damage-specific DNA glycosylases create abasic sites, which are cleaved by APE1, generating SSBs recognised by PARP-1. DNA polymerase β and the XRCC1-DNA ligase IIIα complex fill and seal the gap. DSB repair depends on the cell cycle stage: NHEJ (classical and alternative) is active throughout the cell cycle, with cNHEJ initiated by Ku70/80 binding and recruitment of DNA-PKcs and XRCC4-DNA ligase IV; A-NHEJ involves MRN complex resection, PARP-1 binding, and ligation by DNA ligase I or XRCC1-DNA ligase IIIα. In the S/G2 cell cycle phase, HR repairs DSBs via MRN complex resection, RPA and RAD51 binding, strand invasion by RAD52 and RAD54, and Holliday junction resolution. CDD requires multiple pathways to resolve the lesions, which will depend on the specific nature of the damage. Efficient coordination of these pathways is crucial for the timely repair of PBT-induced damage, promoting cell survival.

The therapeutic efficacy of PBT primarily depends on inducing substantial DNA damage in tumour cells, overwhelming their capacity for repair, and ultimately driving them towards cell death. A variety of DNA lesions can form along the radiation track of the proton beam, including DNA base damage and DNA single-strand breaks (SSBs). However, the most critical lesions contributing to PBT-induced cell death are DNA double-strand breaks (DSBs) and CDD, due to the difficult nature of their repair [43]. These lesions increase in frequency at and around the Bragg peak due to the increasing LET, that causes clustering of the radiation-induced events [44]. There are several important cellular DNA damage response (DDR) pathways that are utilised to resolve the DNA damage induced by PBT (summarised in Figure 2).

Despite the abundance of SSB and DNA base lesions after PBT, they are relatively quick and easy for the cell to repair, with an approximate half-life for accurate repair of several minutes in most cells [45,46,47]. These lesions, including oxidative base damage and abasic sites, are repaired through the base excision repair (BER) pathway [48]. The more complicated the DNA damage, the more challenging the repair and the more harmful these lesions are for the cell. Indeed, DSBs and CDD are lethal lesions induced by PBT, and it is thought that just one unrepaired DSB can be fatal to the cell [44,49]. Cells have evolved two main pathways to repair DSBs: non-homologous end joining (NHEJ) and homologous recombination (HR). NHEJ is active throughout the cell cycle and therefore thought to be the most utilised DSB repair pathway, which can be sub-classified into classical NHEJ (cNHEJ) and alternative NHEJ (aNHEJ), which differ in speed, accuracy of repair, and mechanism [50,51]. Whilst NHEJ enables rapid repair of the lesion, it is also error-prone, as there is no homologous template available during repair, and so there can be small insertions or deletions [52,53]. HR, in contrast, utilises the sister chromatid for more accurate repair and is therefore error-free, although this pathway can only occur in the S and G2 cell cycle phases [44,54].

CDD is defined as two or more DNA lesions within one to two helical turns of the DNA, and like DSBs, is a major contributor to PBT-induced cell death [20]. The nature of the CDD induced by PBT can be generally classified into either non-DSB or DSB clusters and is therefore likely to require both BER and DSB repair pathways. However, the contribution of different DNA repair pathways to the CDD generated by PBT is unclear. Recent research has demonstrated that BER/SSB repair proteins, including PARP-1, PARG, and OGG1 are critical for the repair of DNA damage induced by relatively high-LET protons delivered via the Bragg peak, indicating that this is largely SSB-associated [55,56]. However, there is conflicting evidence in the literature for the increased involvement of either NHEJ or HR in the repair of DSBs induced by protons with increasing LET [21,57,58,59].

Despite the clinical relevance of PBT in UM, the efficiency of the cellular DDR to PBT-induced damage remains surprisingly underexplored. Emerging evidence suggests that variability in treatment response may be influenced by altered levels of key DNA damage-coordinating proteins, some of which are associated with poorer prognosis [59,60]. Downregulation of ATR, or even a complete absence of this protein, has been associated with significantly worse overall survival in UM [60]. ATR is a master protein kinase regulator in response to DSBs and replication fork stabilisation during HR [60]. DNA-PKcs is another crucial kinase involved in facilitating cNHEJ of DNA DSBs, and its gene expression has been shown to be highly expressed in metastatic UM and associated with poor survival [61]. This study also found that low PARP-1 expression could be used to predict better disease-free survival [61]. Collectively, these data suggest there is an important potential role for DNA-PKc and PARP-1 in the initial response to primary UM treatment. Loss of nuclear ATM has additionally been linked with reduced disease-free survival, which is important as ATM is a master regulator kinase involved in DSB repair detection and transduction, cell cycle control, and pathway selection [44,49,62]. Data from our group has recently shown that different UM cell lines show altered expression of ATM, which partly correlates with their response to both X-rays and PBT [63]. Here, high ATM-expressing UM cells were demonstrated to display increased radioresistance driven through enhanced DSB repair rates, compared to lower ATM-expressing cells. Further studies are nevertheless needed to explore the efficiency of the DDR in UM cells, specifically following PBT. Noteworthy is that the majority of studies investigating the role of DDR proteins in mediating ionising radiation-induced cell death have been conducted using X-rays or γ-rays. In contrast, there is a notable lack of evidence elucidating the specific mechanisms underlying PBT-induced cell death. This discrepancy is partly attributable to the limited accessibility of PBT, which requires specialised infrastructure, incurs higher costs, and is subject to geographic disparities compared to more widely available ionising radiation modalities [64]. Nevertheless, given the shared physical and biological principles underlying different forms of ionising radiation, findings from studies using X-rays or other conventional sources are likely to yield comparable insights and should not be disregarded [49]. However, evaluating DNA damage proficiency of UM cells is essential for understanding their response to PBT radiation-induced injury, as any deficiencies or abundance in any of these pathways can significantly influence the therapeutic efficacy of PBT.

### 4.2. The Autophagy Response

**Figure 3:**
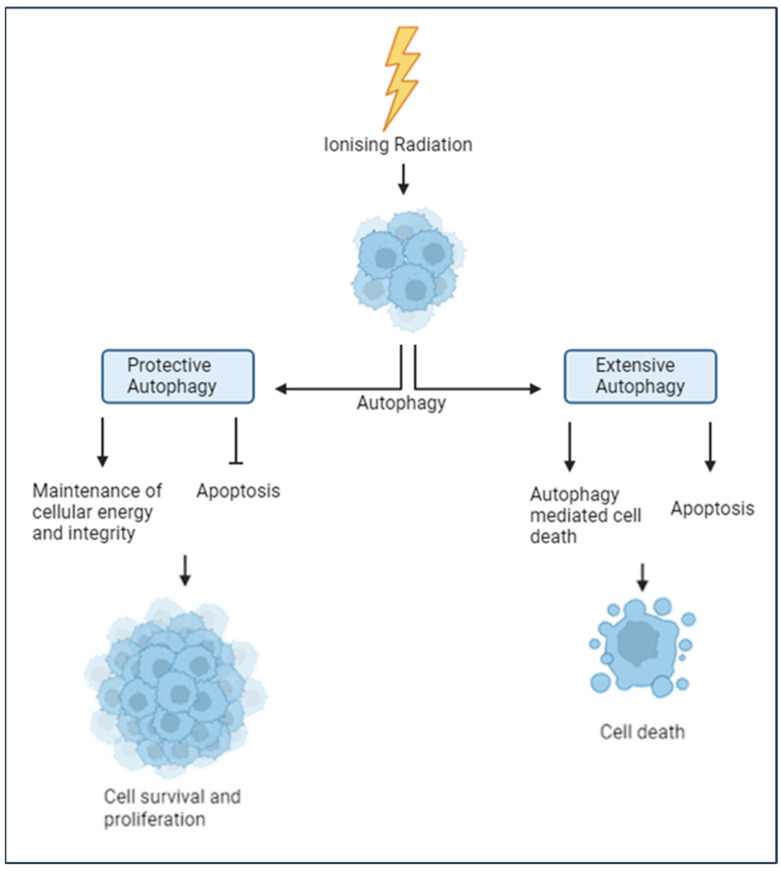
Autophagy can have a major influence on cell fate after ionising radiation. In response to radiation, such as PBT, autophagy can be activated to promote either tumour cell survival or death. Protective autophagy can lead to inhibition of apoptosis and the mitigation of oxidative stress, whilst maintaining the integrity of energy homeostasis. Conversely, extensive autophagy, stimulated through either excessive damage or individual cellular preferences, can lead to uncontrolled degradation of essential cellular components, disrupt homeostasis, and trigger cell death either directly through crosstalk with apoptosis, or indirectly through amplifying necroptosis and autophagy-dependent cell death pathways. The dark blue of the cell represents the nucleus, whereas the lighter blue represents the cytoplasm. Image created in BioRender. Hawkins, L. (2025) https://app.biorender.com/illustrations/67373569041045f9a52893c7?slideId=9ffb5c00-6e63-43ff-8466-b9f117882195 (accessed on 13 September 2025).

Autophagy is a highly conserved process responsible for breaking down damaged macromolecules, cellular structures, and organelles through lysosomal degradation to maintain cellular homeostasis, promoting either cell survival or cell death (Figure 3) [65,66]. Ionising radiation, including PBT, also increases reactive oxygen species (ROS) levels, impairing mitochondrial function, causing protein misfolding, and triggering the release of calcium from the endoplasmic reticulum to the cytoplasm, all of which are factors shown to induce autophagy [65,67]. Cancer cells exploit autophagy pathways to enhance stress tolerance and act as a protective mechanism against therapeutics, including ionising radiation [67,68]. Autophagy can act as a cryoprotective mechanism against PBT-induced stress through the clearing of damaged organelles and proteins, the maintenance of cellular energy levels, and the modulation of apoptotic pathways [69,70]. However, if cellular damage and metabolic stress exceed the cellular repair capacity, extensive autophagic flux can lead to accelerated cell death. Autophagy-driven cell death may occur directly or involve other key cell death pathways, such as apoptosis or necrosis, particularly under severe damage [71,72]. Therefore, the cellular balance of protective autophagy compared to extensive autophagy is likely to play a huge role in the impact of PBT damage and the cellular response.

Precisely how autophagy mediates and influences the impact of PBT and the cellular response in UM remains largely unclear. It has been suggested that autophagy plays an important role in UM cells, as it has been estimated that these cells increase autophagic activity by around 40–50% above basal levels in their non-cancerous counterparts [73]. This increase in autophagy activity could be a byproduct of the hypoxia-induced metabolic stress in tumour cells or may suggest that autophagy offers significant survival and tumorigenic advantages. Evidence indicates that autophagy may play an actively protective role in UM, as shown with the inhibition of autophagy with chloroquine, which led to enhanced apoptosis and increased autophagic flux (64). Chloroquine has also been shown to sensitise UM cells and tumours harbouring GNAQ/GNA11 mutations, which are present in over 90% of UM cases, to the MEK1/2 inhibitor trametinib [74]. Although data remain limited, existing evidence suggests that UM cells may exhibit elevated autophagy activity and are partially dependent on its protective functions, such as inhibiting apoptosis, preserving energy stores, and maintaining cellular integrity, to support tumour cell survival under normal conditions. This reliance on the protective mechanisms of autophagy could reduce the effectiveness of PBT in some UM, as enhanced autophagic responses may help cells better withstand the stress induced by the radiation. However, it is currently impossible to rule out the dual roles that autophagy may play in the biological effect of PBT. To fully understand the therapeutic implications, further research is needed, especially with PBT.

### 4.3. Hypoxia and the Tumour Microenvironment

Hypoxia is defined as the reduction of oxygen in tissues and is often the result of the metabolic demands of the growing tumour exceeding the supply of oxygen [75]. Reductions in cellular oxygen levels can also strongly impact the effectiveness of radiotherapy [76,77]. Under normal physiological conditions, ionising radiation reacts with molecular oxygen in the tissue, producing ROS, which in turn cause further damage to vital cellular components such as DNA and organelles [76]. Molecular oxygen also enables the creation of fixed peroxyl adducts onto the DNA structure, which are incredibly difficult for the cell to repair, termed the “oxygen fixation hypothesis”, and are dangerous if these lesions are carried through to DNA replication [76,78]. During hypoxia, less molecular oxygen is available as a substrate for ROS generation, resulting in decreased DNA damage, theoretically reducing the efficacy of PBT in hypoxic tumours. Therefore, the levels of oxygen in the tumour at the time of PBT are a crucial factor in the biological response of this treatment.

A hypoxic microenvironment is an important feature of UM and is strongly associated with malignant progression, tumour aggressiveness, and reduced overall survival [75,79]. Studies have shown that hypoxia correlates with elevated genomic instability and *BAP1* mutations, resulting in promotion of cell survival through cell cycle checkpoint interference and less favourable clinical outcomes [75,80]. The development of a hypoxic tumour microenvironment is also associated with modulation of the immune response and results in reduced immune cell infiltration, due to newly formed leaky blood vessels, which further enables cell proliferation and metastasis [75,81]. The adaptation to hypoxia is regulated by hypoxia-induced-factors (HIF), where HIF-1α stabilisation is a crucial early event in the cellular response to hypoxia [82,83]. HIF-1α protein is reportedly highly expressed in UM and supports cell survival through regulation of DNA damage pathways, cellular metabolism, apoptosis, and autophagy [75,84]. The cellular and metabolic response to PBT in a hypoxic cell compared to a normoxic cell may be different based on these transcriptional changes and enrichment of pro-survival pathways. However, the impact that this has on the efficacy of PBT, and importantly how this can be harnessed to enhance treatment effectiveness, is not known.

## 5. Strategies to Exacerbating the PBT Response in UM

There is an urgent need to explore options to further enhance the radiotherapeutic response to PBT in UM in a safe manner, as current treatments can cause unpleasant adverse effects, such as toxic tumour syndrome and radiation retinopathy, which greatly diminish quality of life [33,35,85]. Some primary tumours also do not respond well to treatment or relapse and reoccur shortly after PBT, events which are associated with a poorer prognosis and increased tendency to metastasise [86]. The most promising approaches to improve the radiobiological effectiveness of PBT in UM are evaluated below.

### 5.1. Targeting DNA Damage Repair

**Figure 4:**
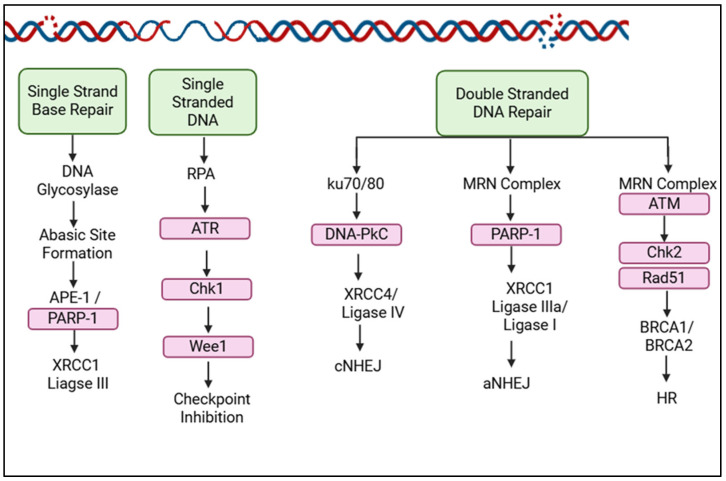
Cellular proteins within the DDR currently under investigation for drug/inhibitors to enhance radiotherapy efficacy. The major repair proteins within the key pathways responsible for co-ordinating the signalling and/or repair of DNA damage induced by PBT are shown. The proteins highlighted in pink have been studied as radiosensitisation targets, and inhibitors have been developed against them with the aim to further increasing the cellular response to radiation. Created in BioRender. Hawkins, L. (2025) https://app.biorender.com/illustrations/662e6ad9a3cc858692ca7beb?slideId=c85d73e2-92cc-40d6-a481-b6f1809d2103 (accessed on 13 September 2025).

Given that the primary therapeutic effect of PBT relies on inducing DNA damage, targeting the DDR with specific inhibitors represents a promising strategy to enhance treatment efficacy and improve patient outcomes. Some of the major cellular targets for drugs and inhibitors that have been developed to date are shown below (Figure 4).

Inhibiting the proteins governing the repair of lethal lesions such as DSBs and CDD is a promising therapeutic strategy to sensitise UM cells to PBT [21,60,87]. These include the three major protein kinases ATM, DNA-PKcs, and ATR, which are involved in the signalling and coordination of the DDR [63,88]. Recent evidence indicated that targeting ATM with a potent and specific inhibitor can increase the radiosensitivity of four different UM cell lines with different genetic backgrounds to both X-rays and PBT, mediated through inhibition of DSB repair [63]. Inhibition of DNA-PKcs has also been demonstrated to radiosensitise UM cell lines and xenograft mouse models to γ-radiation [89,90]; theoretically, this radiosentisation effect could also apply to the radiation-induced damage caused by PBT.

PARP inhibitors are another class of promising radiosensitisers and have already been approved for the treatment of BRCA-mutated breast and ovarian cancers [91]. Inhibiting this key SSB repair protein results in increased DSBs formed through the collapse of the DNA replication forks, which are particularly toxic to cells with HR deficiency, a concept known as ‘synthetic lethality’ [92,93]. In UM, HR deficiency is not usually present [89,90]; however, there is a growing amount of research that suggests that some genetic alterations, such as loss of *BAP1* or the *SF3B1* hotspot mutations, may confer sensitivity to PARP inhibitors [94,95,96]. The combination of PARP inhibitors plus radiation, particularly PBT, therefore requires further research to determine potential synergistic effects. Combining the precision of PBT with the favourable toxicity profile of PARP inhibitors presents a particularly promising therapeutic strategy, potentially offering enhanced radiobiological effects with minimal adverse outcomes.

Nevertheless, evidence examining the impact of DDR inhibitors to radiosensitise UM models is very limited. Investigating this approach is crucial, as enhancing the radiobiological effects of PBT by disrupting efficient DNA repair could have significant clinical implications, such as enabling lower radiation doses to minimise side effects or expanding treatment options for larger tumours, potentially preserving the eye and vision.

### 5.2. Other Potential Radiosensitisation Targets

Since autophagy can either support cell survival or promote cell death, its role in enhancing the efficacy of PBT in UM remains uncertain, making it a challenging therapeutic target. One study has reported that elaiophylin, an inhibitor of autophagosome formation, has demonstrated potential as a monotherapy in UM cell lines, inducing autophagic cell death via mitophagy suppression and the accumulation of oxidative stress [97]. Chloroquine and hydroxychloroquine, as autophagy inhibitors, have been investigated in other tumour types, but with no clear clinical benefit established [98]. mTOR inhibition, a central negative regulator of autophagy, has been explored as a strategy to promote autophagy [99,100]. Rapamycin is a potent mTOR inhibitor that has shown some potential in enhancing tumour cell responses to radiation by increasing autophagic activity [101]. However, the lack of reliable biomarkers to predict patient responses to autophagy agents further complicates their clinical utility. Regardless, it would be interesting to examine whether modulating autophagy through these promising targets could increase the effect of radiation in UM cells and tissues, and their subsequent response, particularly with targeted PBT.

Overcoming radioresistance caused by tumour hypoxia is another strategy to promptly drive an increased PBT response. An early approach used to achieve this was through carbogen to directly increase the oxygen inside the tumour, although this may paradoxically increase the sensitivity of healthy tissues to radiation, potentially worsening adverse side effects [102]. More recent strategies have focussed on inhibiting key proteins involved in the hypoxic response, such as HIF1α, which is a promising target in UM as it is known to be overexpressed in metastatic tumours [84]. An inhibitor of HIF1α in mouse models has been shown to suppress UM primary tumour growth, liver metastasis, and subsequent survival as a monotherapy whilst being well tolerated [103]. Unfortunately, there is no further evidence in UM to investigate HIF1α as a target for radiosensitisation. Additionally, it is thought that HIF2α is involved in more of a chronic response to hypoxia, whereas HIF1α rapidly mediates the acute response [104,105]. Therefore, investigating HIF2α as a target in UM is also a potential strategy that warrants future research. Targeting the hypoxic response presents a promising strategy to enhance the effectiveness of PBT, as alleviating hypoxia could increase DNA damage and diminish the cell’s ability to manage radiation-induced stress. By disrupting this adaptive survival mechanism, tumour cells may become more vulnerable to treatment, potentially leading to improved therapeutic outcomes.

### 5.3. Radiotherapy Advances

Advances in treatment planning and delivery of precision-targeted PBT could further improve the outcomes of UM treatment whilst still sparing the eye. The efficacy of PBT is highly dependent on the accurate positioning of the Bragg peak to the tumour, although issues such as complex dose calculations and limited optimisation algorithms hinder this process [106]. Advancing already established radiotherapy techniques is another method to improve treatment for UM patients. This includes stereotactic radiosurgery using the Gamma Knife (GK-SRS), an alternative globe-sparing radiotherapy for UM. A large systematic analysis found that this GK-SRS treatment is as effective as PBT in achieving five-year survival rates [107]. While GK-SRS offers similar local tumour control to PBT, it is associated with higher rates of adverse side effects, including vitreous haemorrhage and secondary glaucoma, occurring in up to 47% of patients [108,109]. An in-silico study comparing GK-SRS and PBT found that, whilst both therapies offer precise tumour targeting, PBT provides better retina sparing and reduces toxicity, particularly haemorrhage [108]. This underscores the advanced precision of PBT and the ongoing challenge of optimising its delivery in UM treatment.

### 5.4. FLASH Radiotherapy

FLASH radiotherapy, characterised by ultra-high dose rates (>40 Gy/s), has emerged as a promising technique to reduce normal tissue toxicity while maintaining effective tumour control [110]. This approach is particularly relevant for tumours such as UM, which are typically small, thin lesions located near critical structures like the optic nerve and the retinal pigment epithelium (RPE) [85]. Combining the precision of PBT with the ability of FLASH delivery to spare surrounding healthy tissue could offer a powerful alternative to further reduce adverse side effects in UM treatment. Although PBT is highly accurate, it can still cause vision-threatening complications, including radiation retinopathy and optic neuropathy (as shown in Table 1). FLASH radiotherapy may help protect the RPE during UM treatment, which is essential for maintaining the survival of photoreceptors crucial for vision [85]. Recent studies have demonstrated that FLASH treatment enhances the viability of ARPE-19 cells and results in a milder decline in motor function in treated mice compared to conventional irradiation [111].

However, preclinical and clinical data on the use of FLASH in ocular applications remain limited. Neither FLASH nor conventional irradiation was shown to affect depth perception in a mouse model, although the radiation dose was delivered to the brain, not the eye, which may explain the lack of sensitivity in the assay [111]. Moreover, the development of FLASH was not ranked as a high priority in the 2022 Second International Survey of ocular proton centres. Instead, increased access to PBT and the creation of a commercially available, sustainable treatment planning system were considered more critical for ongoing development [112]. So, while FLASH PBT shows promise for UM treatment and early data are generally encouraging, further research is needed to fully explore its potential in mitigating the adverse side effects associated with PBT in UM.

## 6. Concluding Remarks and Future Directions

UM is a sight-threatening and often fatal intraocular cancer. Primary treatment with PBT is effective, resulting in high local control and overall survival rates of ~80–90%. However, some patients still experience poor outcomes due to treatment resistance, relapse, or metastasis. Whilst the precision of PBT allows for the treatment of tumours regardless of size or location within the eye, the biological factors influencing differential responses are not yet fully understood. Emerging evidence suggests that cellular mechanisms such as the DDR, tumour hypoxia, and autophagy play important roles in modulating the effectiveness of radiotherapy. Yet, there is a surprising lack of data identifying the specific proteins and pathways involved in the response of UM to PBT. This gap in knowledge limits our ability to optimise treatment strategies and personalise therapy. Future research should focus on dissecting the molecular landscape of UM using both immortalised and patient-derived cell models with diverse genetic backgrounds to better understand how genetic and cellular processes influence radiation sensitivity. In particular, exploring the potential of DDR inhibitors, hypoxia-targeting agents, and autophagy modulators in combination with PBT could pave the way for more effective, tailored therapeutic approaches that improve patient outcomes while minimising the significant adverse effects.

It is accepted that the most lethal lesions induced by PBT are DSBs and CDD. Therefore, targeting the DDR is considered a primary strategy through which radiotherapy effectiveness could be enhanced. Whilst not studied in great depth, there is some evidence to suggest that ATM and DNA-PKc inhibition can increase the sensitivity of UM models to PBT. However, there is also the potential for the use of autophagy or hypoxia modulators to enhance cell death after radiation treatment. Despite this, there is generally limited data for the application of these treatment strategies in UM to enhance the PBT response, where more extensive preclinical research is needed to identify their potential. These strategies need to be explored in vitro, using both cultured cell lines and also more advanced models including patient-derived organoids, as well as in vivo using the appropriate animal models. Such research is vital for identifying strategies in enhancing the effectiveness of PBT in the treatment of patients with UM, contributing towards improving patient outcomes and quality of life. This could also lead to preventing tumour recurrence and/or fatal liver metastasis.

## Figures and Tables

**Table 1:** A table of reviews evaluating UM patient outcomes after PBT.

Time	Patient Numbers	Dose	Median Follow up	Local Control	Eye Preservation	Overall Survival	Toxicity	Ref
1984–1999	2648	60 Gy in 5 fractions	24 months	5-year 95.8% 10-year 94.8%	91.8%	10-year 73%		[29]
1993–2003	349	53.1 Gy in 4 fractions	37 months	5-year 96.5%	92%	5-year 90%	17.9% glaucoma 12.8% rubeosis 16.7% pain 9% vitreous haemorrhage 38% retinal detachment	[30]
1991–2001	1406	60 Gy in 4 fractions	73 months	5-year 96%	92.3%	5-year 79%	66.5% maculopathy 23.4% papillopathy 28.6% glaucoma 61.8% cataract 11.5% keratitis 13.9% vitreous haemorrhage	[18]
1991–2007	886	60 Gy in 4 fractions	64 months	5-year 93.9% 10-year 92.1%	92.2%	5-year 79.4% 10-year 64.1%	7% glaucoma 27.5% retinopathy 31.7% cataract	[31]
1993–2008	147	58 Gy in 4 fractions	53 months		5-year 71.3%	5-year 87.8%		[32]
1995–2007	59	60 Gy in 4 fractions	125 months	5-year 91%	5-year 93.2%	5-year 85%	74% radiation retinopathy 64% optic nerve neuropathy 29.6% cataract	[33]
1998–2008	982	60 Gy in 4 fractions	60.7 months	5-year 96% 10-year 94%	5-year 95%	5-year 80% 10-year 60%	12.1% neovascular glaucoma	[34]
1991–2015	492	60 Gy in 4 fractions	61.9 months	5-year 60% 10-year 47%	80.5%		39.8% cataracts 27% neovascular glaucoma 23.4% retinopathy	[35]
